# Alcohol Consumption Patterns and Psychological Pain Among Patients Hospitalized After Self-Harm

**DOI:** 10.1192/j.eurpsy.2026.10689

**Published:** 2026-07-31

**Authors:** M. Fjodorova, J. Manuhina, M. Gebele, J. Vrublevska

**Affiliations:** 1 Riga Stradins University, Riga; 2 Hospital Gintermuiza, Jelgava; 3 Faculty of Medicine and Life Sciences, Department of Neuromedicine and Neurosciences, University of Latvia, Riga, Latvia

## Abstract

**Introduction:**

Self-harm is a complex multifactorial issue that involves various aspects, including psychiatric, psychological and social factors, which influence person’s propensity to engage in self-harm. Maladaptive coping strategies, such as alcohol use and self-harm, are often employed as attempts to escape from unbearable psychological pain.

**Objectives:**

To identify risk factors including alcohol consumption habits and psychological pain in patients admitted to emergency department following self-harm episode. To analyse association between alcohol consumption and psychological pain.

**Methods:**

A hospital based, cross-sectional study was performed among patients admitted to regional psychiatric hospital Gintermuiža from 01.11.2024. to 06.09.2025. after self-harm episode and consented to participate in the study. Socio-demographic, clinical data, frequency of alcohol use, amount consumed per occasion, alcohol use during self-harm and Visual Analogue Scale (VAS) for the level of psychological pain were used.

**Results:**

The study included 61 patients aged 18 to 86 years. Median age 38 years. 50.8% (n=31) women and 49.2% (n=30) men. 23% (n=14) did not consume alcohol at all, 32.8% (n =20) patients reported consuming alcohol once a month or less, 26.2% (n=16) consumed alcohol 1-4 times per month, 11.5% (n=7) consumed alcohol 2-3 times per week, and 6.6% (n=4) consumed alcohol more than 4 times per week. 37.7% (n = 23) reported consuming alcohol at the time of self-harm. The mean psychological pain score at admission time was 4.93 (SD =2.77) on a 10-point scale. 8.2% (n=5) reported no psychological pain (VAS=0), 16.4% (n=10) reported mild psychological pain (VAS=1-3), 44.3% (n=27) reported moderate psychological pain (VAS=4-6), 23% (n=14) reported severe psychological pain (VAS=7-9) and 8.2% (n=5) worse pain (VAS=10). No statistically significant correlation found between the frequency of alcohol consumption and psychological pain level (p=0.702). No statistically significant correlation found between the amount of alcohol consumed per occasion and the level of psychological pain (ρ=–0.011; p=0.935). There was found no significant difference in psychological pain between participants who used alcohol during self-harm and those who did not (p=0.214).

**Conclusions:**

There was found no association between alcohol consumption patterns and psychological pain among patients with self-harm behavior. Psychological pain represents an independent factor. This might be explained by the fact that psychological pain is highly subjective measure influenced by personal experience, stress tolerance and cognitive factors. The relatively small cohort represents a limitation of this study, limiting the statistical power to detect smaller effects and highlighting the need for further investigation with a larger sample size.

**Disclosure of Interest:**

None Declared

